# Supplementary material for: Tunable exciton valley-pseudospin orders in moiré superlattices
Source: Nat Commun. 2024 May 18;15:4254. doi: 10.1038/s41467-024-48725-z (PMC11102517; doi:10.1038/s41467-024-48725-z)
Supplement: Supplementary file 1 — Supplementary Information [file 41467_2024_48725_MOESM1_ESM.pdf]

Supplementary Information for "Tunable exciton valley-pseudospin orders in moiré superlattices"

Richen Xiong[1], Samuel L. Brantly[1], Kaixiang Su[1], Jacob H. Nie[1], Zihan Zhang[1], Rounak Banerjee[2], Hayley Ruddick[2], Kenji Watanabe[3], Takashi Taniguchi[4], Sefaattin Tongay[2], Cenke Xu[1], Chenhao Jin[1]*

[1]*Department of Physics, University of California at Santa Barbara, Santa Barbara, CA, USA*

[2]*School for Engineering of Matter, Transport, and Energy, Arizona State University, Tempe, AZ, USA.*

[3]*Research Center for Functional Materials, National Institute for Materials Science, Tsukuba, Japan.*

[4]*International Center for Materials Nanoarchitectonics, National Institute for Materials Science, Tsukuba, Japan*

* Corresponding author. Email: jinchenhao@ucsb.edu

Contents:

Supplementary Figure 1. Basic characterizations of devices.

Supplementary Figure 2. Response of a bosonic correlated insulator to transient extra K excitons.

Supplementary Figure 3. Optical characterization of device D2.

Supplementary Figure 4. Probe-induced spin imbalance in device D2.

Supplementary Figure 5. Polarization-resolved probe-induced PL spectra of device D1 at 60 K.

Supplementary Figure 6. Probe-induced spin imbalance in device D1 at 60 K.

Supplementary Figure 7. Normalized generalized helicity and phase diagrams of Δ_{GH} at $B_z = 30$ mT.

Supplementary Figure 8. Linear polarization-resolved PL at $\nu_{\mathrm{ex}} = 1.1$.

Supplementary Figure 9. Estimation of measurement uncertainties.

Supplementary Figure 10. Calibration of exciton density via TRPL measurement.

Supplementary Note 1. Mean field analysis of a phenomenological spin model

Supplementary Figures:

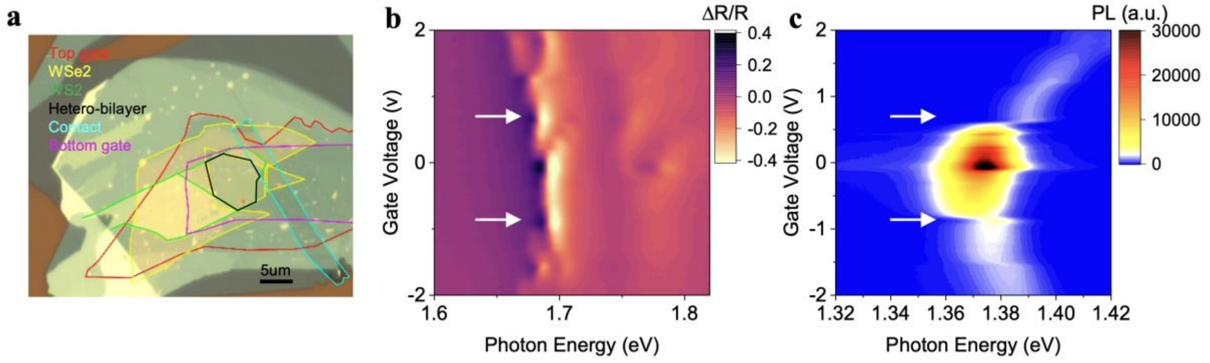

Supplementary Fig. 1: Basic characterizations of devices. **a**, optical image of a representative dual-gated 0-degree-aligned WSe_2/WS_2 device D1. Yellow and green solid lines denote contours of the monolayer WSe_2 and WS_2 flakes, respectively. **b,c,** Electron doping-dependent absorption (**b**) and PL (**c**) spectrum of device D1 at zero pump intensity. White arrows indicate one filling of electrons and holes.

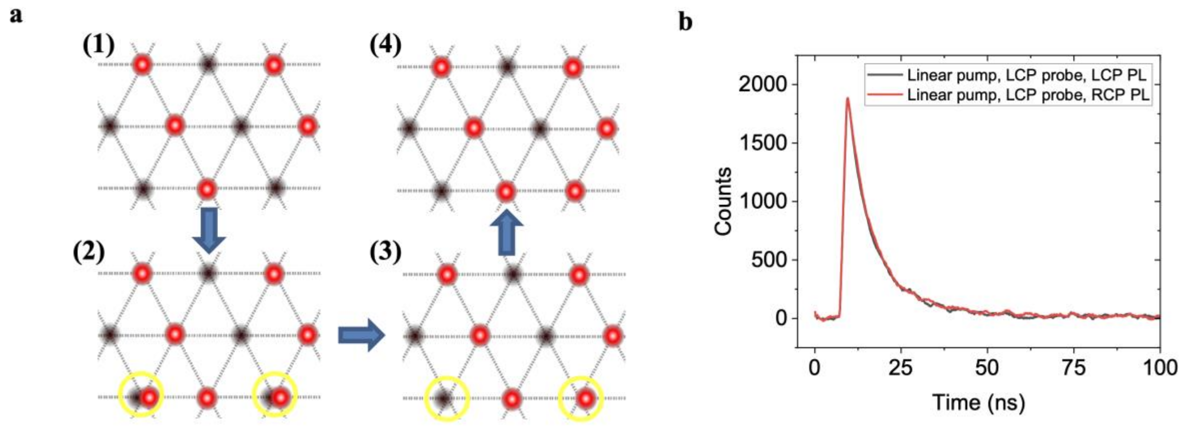

Supplementary Fig. 2: Response of a bosonic correlated insulator to transient extra *K* excitons. a, (1), CW linear pump light injects equal number of *K* valley (red) and *K'* valley (grey) background excitons that form an exciton lattice. **(2),** Pulsed circular polarized probe light transiently injects two extra *K* valley excitons, which takes two *K'* sites to form doublon sites (yellow circles). **(3)**, Doublon sites have equal probabilities to emit *K*(*K'*) excitons and leave a single site of *K'*(*K*). **(4)**, After the doublons decay the system will have one more *K* single site and one less *K'* single site, giving rise to negative *K'* response and positive *K* response of peak I with equal amplitude. **b,** Probe-induced TRPL measurements of doublon emissions under linear pump and LCP probe configuration. The two valleys show identical amplitude and dynamics, consistent with the expectation of doublon emission.

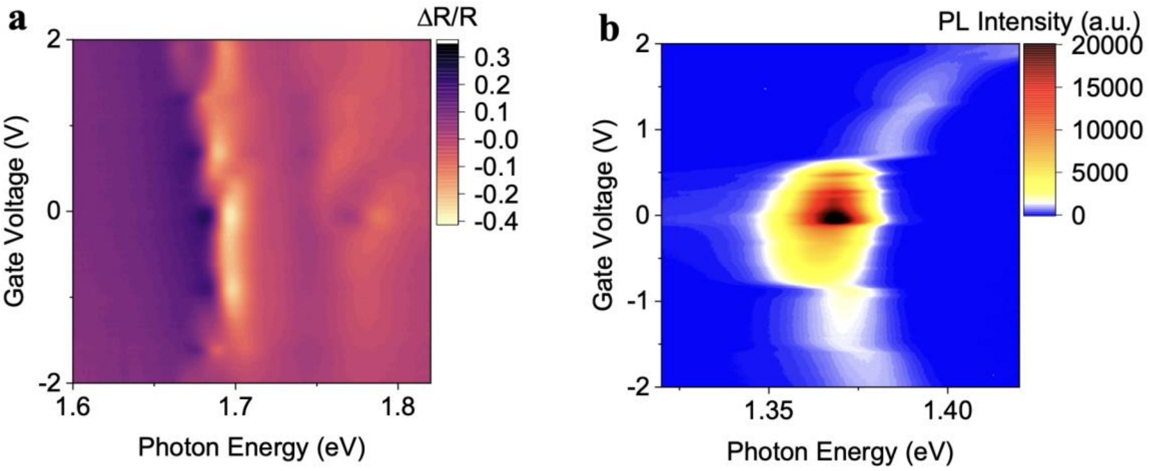

Supplementary Fig. 3: Optical characterization of device D2. Doping-dependent absorption (**a**) and PL (**b**) spectrum of the 0-degree aligned WSe_2/WS_2 device D2 at zero pump intensity.

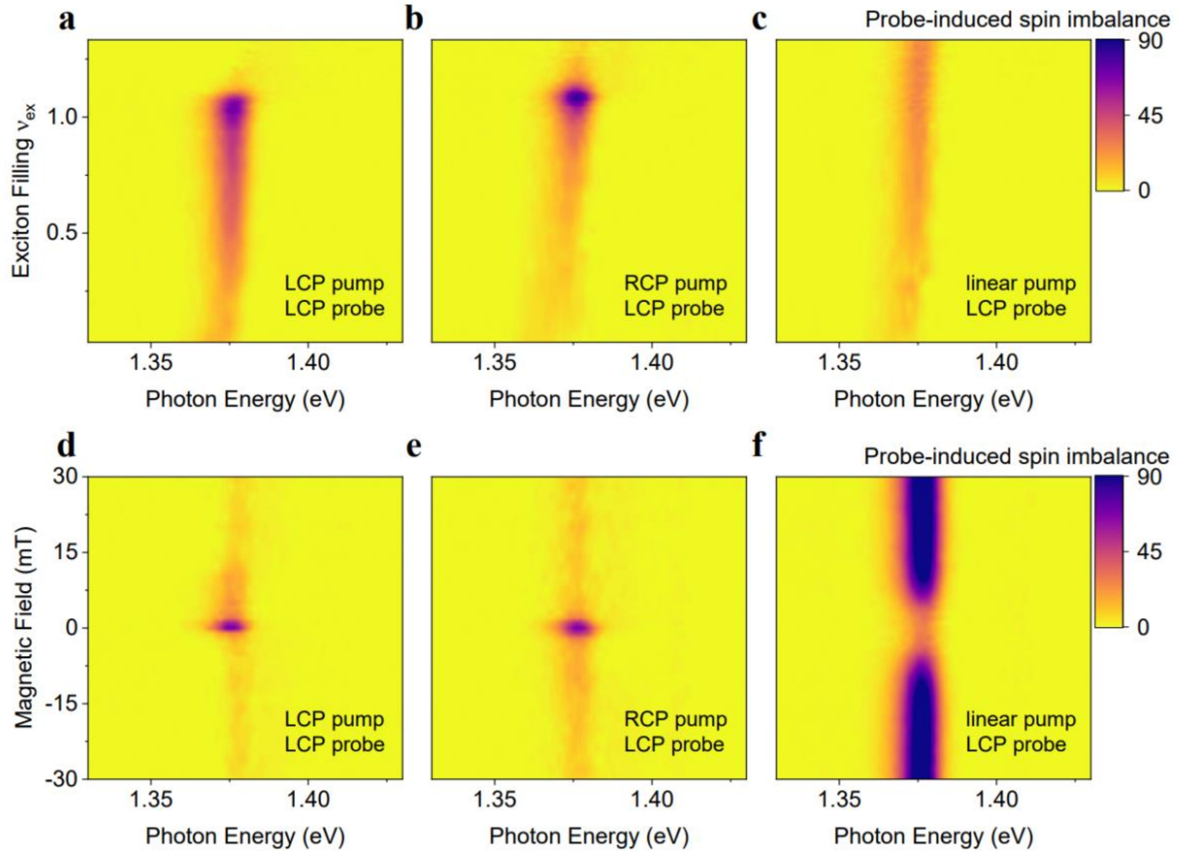

Supplementary Fig. 4: Probe-induced spin imbalance in device D2. a-c, LCP probe-induced spin imbalance (arbitrary units) as a function of background exciton fillings for LCP (**a**), RCP (**b**) and linear (**c**) pump respectively. **d-f,** Evolution of probe-induced spin imbalance (arbitrary units) signal at ν_{ex}=1.1 under out-of-plane magnetic field B_z for LCP (**d**), RCP (**e**) and linear (**f**) pump, respectively.

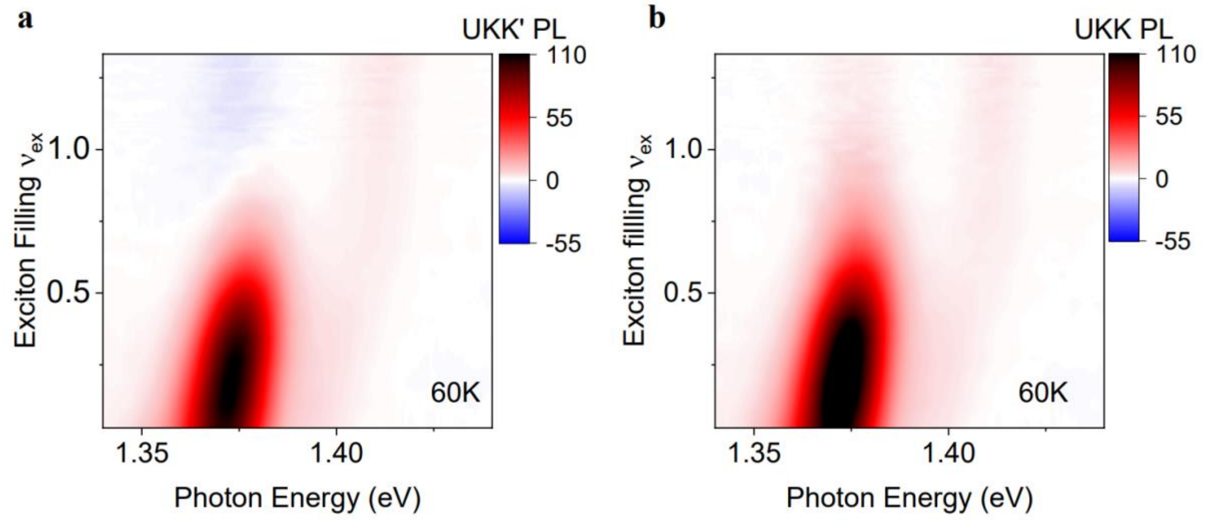

Supplementary Fig. 5: Polarization-resolved probe-induced PL spectra of device D1 at 60 K. A linear pump light is used to generate equal numbers of *K* and *K'* valley excitons in the background, while an LCP probe light selectively excites extra *K* valley excitons. *K'* valley (**a**) or *K* valley (**b**) PL response induced by the probe light is collected separately. The bosonic correlated insulator state and on-site AFM interaction are still robust at 60 K. UKK (UKK') refers to pump injecting unpolarized excitons, probe injecting *K* valley excitons and PL detecting *K* (*K'*) valley excitons.

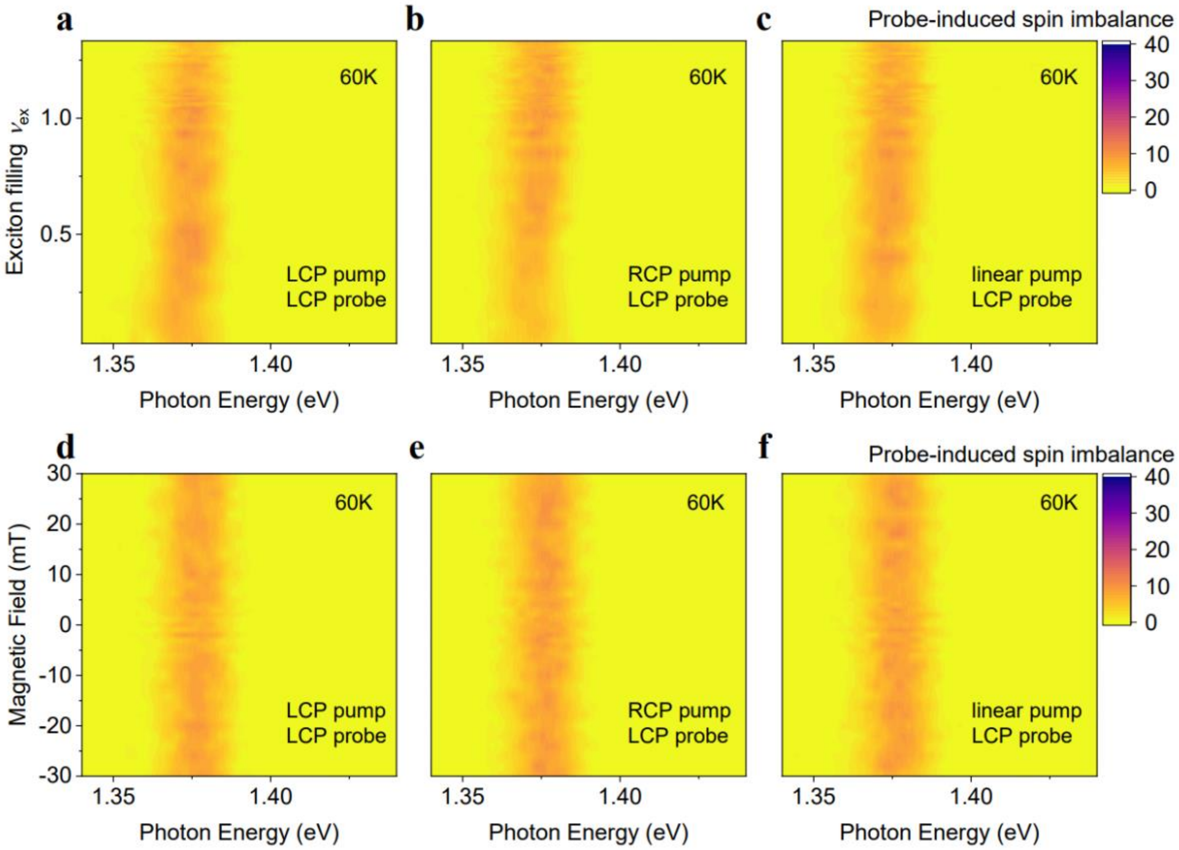

Supplementary Fig. 6: Probe-induced spin imbalance in device D1 at 60 K. a-c, Probe-induced spin imbalance (arbitrary units) at 60 K as a function of background exciton fillings for LCP (**a**), RCP (**b**) and linear (**c**) pump respectively. **d-f,** Evolution of probe-induced spin imbalance (arbitrary units) at ν_{ex}~1.1 and 60 K under out-of-plane magnetic field B_z for LCP (**d**), RCP (**e**) and linear (**f**) pump, respectively. No pump polarization dependence is observed across all exciton fillings, indicating vanishing spin-dependent interactions at 60 K.

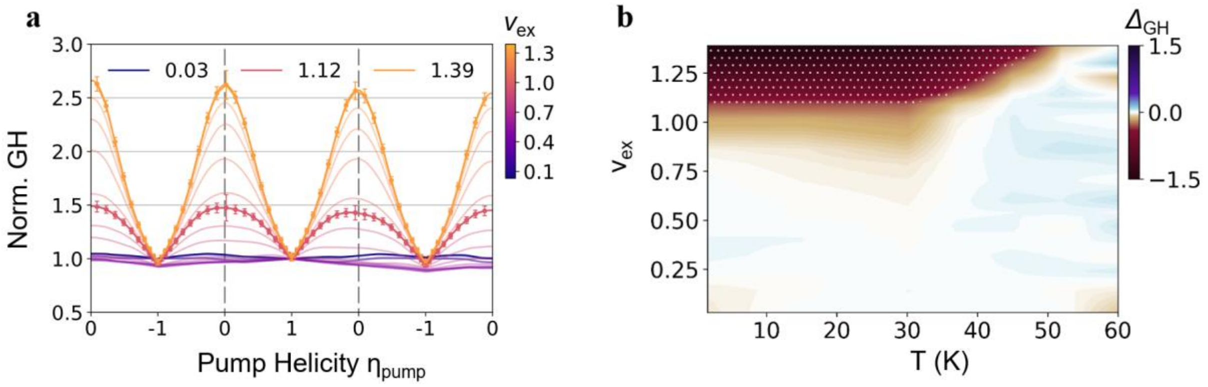

Supplementary Fig. 7: Pump–only PL measurement at B_z= 30 mT. a, Normalized generalized helicity (GH) at B_z= 30 mT. Error bars represent standard deviation in normalized GH. **b,** Phase diagrams of Δ_{GH} at B_z= 30 mT.

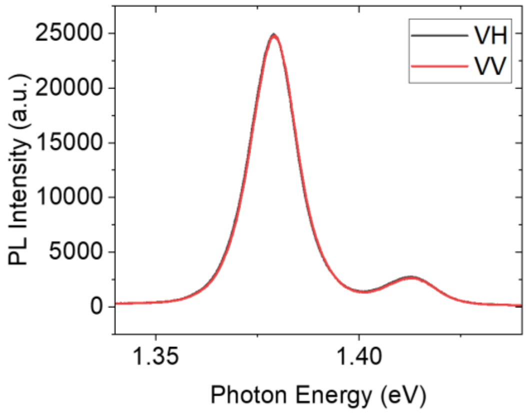

Supplementary Fig. 8: Linear polarization-resolved PL at ν_{ex} = 1.1. No linear helicity is observed between vertical (VV) and horizontal (VH) PL detection, indicating that there is no global long-range FM-*xy* order. The pump light is linearly polarized along the vertical direction.

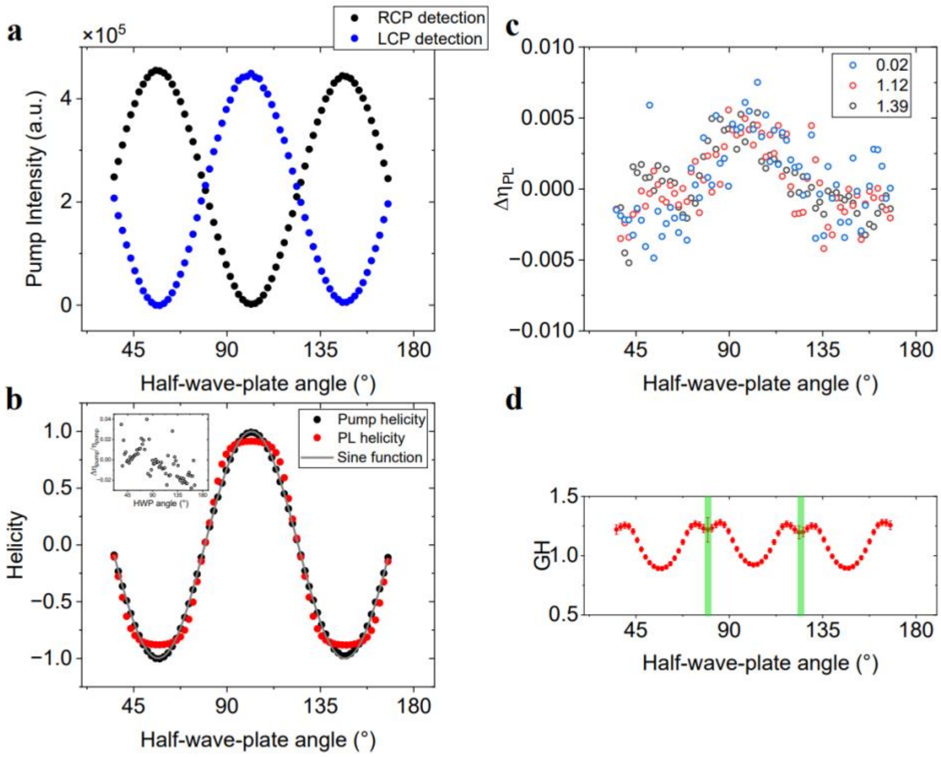

Supplementary Fig. 9: Estimation of measurement uncertainties. a, LCP (blue) and RCP (black) components of the sample–reflected pump light under identical experimental configuration as polarization-resolved PL measurements. **b,** Pump helicity (black symbols) and PL helicity (red symbols) at ν_{ex}=1.39 and zero magnetic field with different HWP angles. The pump helicity shows near-perfect match with theoretical curve (grey line) with a relative standard deviation of 1.7%. Inset shows the deviation between the measured and theoretical pump helicity. **c,** The deviation in η_{PL} between two successive measurements, from which we calculate the standard deviation in η_{PL} to be 0.21%, 0.17% and 0.17% for ν_{ex}=0.02, 1.12 and 1.39, respectively. **d,** Generalized helicity (GH) at ν_{ex}=1.39 and zero magnetic field as a function of HWP angles. The standard deviation (error bars) in GH becomes reasonably small (<5%) when $|\eta_{\mathrm{pump}}|$>0.05 (outside of the green shaded region).

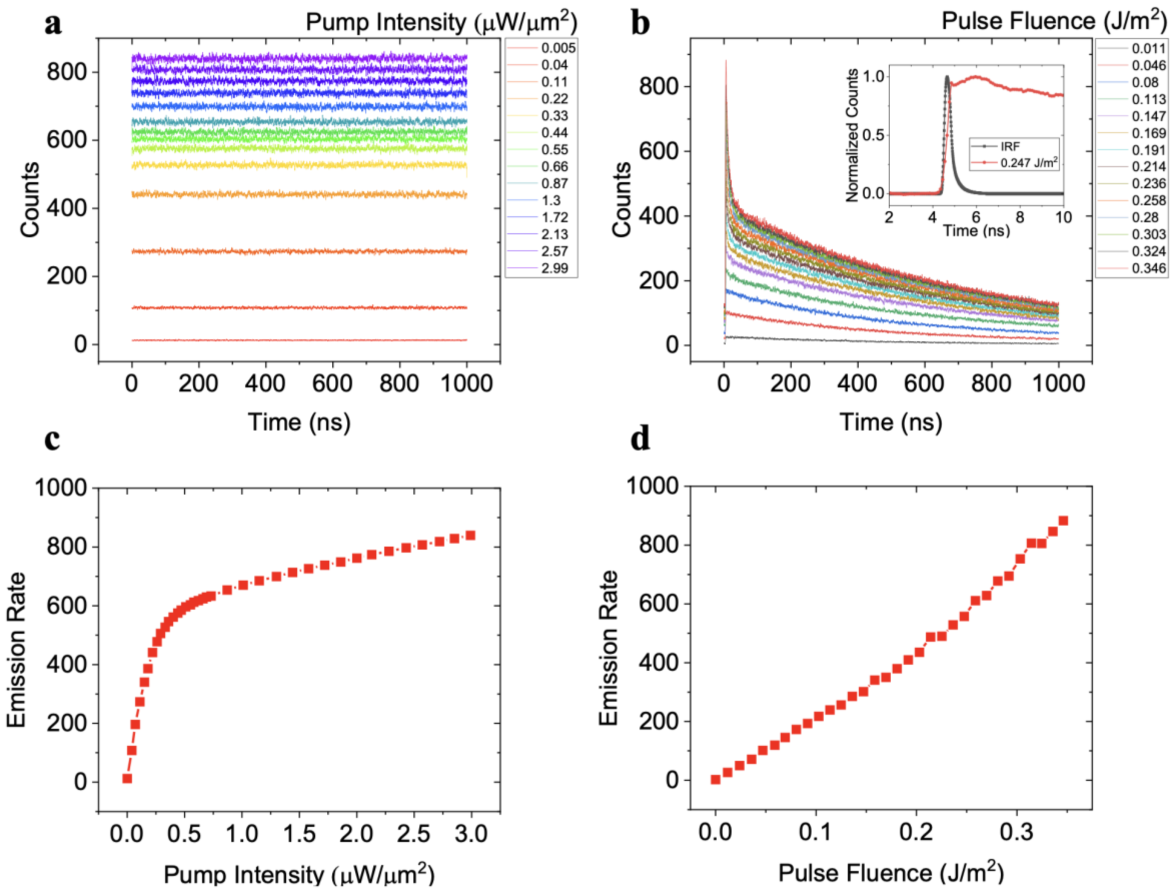

Supplementary Fig. 10: Calibration of exciton density via TRPL measurement. **a,** TRPL using a 660 nm CW pump light of different pump intensity. **b,** Same as **a** but with a 660 nm pulsed pump light (300 ps duration, 1 MHz repetition rate). Inset: Comparison between IRF and PL dynamics indicates negligible exciton relaxation immediately after time zero. **c,** Emission rates from the CW pump light of different intensities. **d,** Emission rates from the pulsed pump light of different fluences.

Suppelementary Note 1: Mean Field analysis of a phenomenological spin model

I. HAMILTONIAN ON THE MOIRÉ SUPERLATTICE

We would like to first write down the Hamiltonian for interacting excitons on the moiré superlattice. The symmetry of the moiré lattice should include P_x which takes $x \to -x$, time-reversal $\mathcal{T}$, and three fold rotation $R_{2\pi/3}$. The P_x and $\mathcal{T}$ both interchanges the two species (pseudo-spin flavors) of the excitons, as the exciton is formed with electron-hole pair from the K and K' valleys respectively. The symmetry allowed Hamiltonian reads

$$H = \sum_{<i,j>,\alpha} -te^{\mathrm{i}\phi_{ij}\tau^{\alpha}} b^{\dagger}_{i,\alpha} b_{j,\alpha} + H.c. + \sum_{i,\alpha} U(\hat{n}_{i,\alpha} - 1/2)^2 + \sum_{i} V \hat{n}_{i,1}\hat{n}_{i,2}. \cdots \tag{S1}$$

The interactions between the excitons include an usual intra-species repulsion U as well as an inter-species repulsion V. Naturally we expect $U > V > 0$.

The symmetry of the system allows the two specicies of excitons to see opposite fluxes through each triangular plaquette of the moiré lattice, and the phase angle of the hopping amplitudes of the excitons satisfy $\tau^1 = -\tau^2 = \pm 1$, and ϕ_{ij} changes sign under rotation $\pi/3$, hence ϕ_{ij} would vanish if the system had a six fold rotation symmetry. The fluxes of the excitons can affect the effective pseudo-spin model arising from superexchanges. If there were no flux, it is well-known that, at precisely filling $\nu_{ex} = 1$ (one exciton boson per moiré unit cell), the pseudo-spin physics of the system is captured by the following XXZ model in the limit $U > V \gg t$ [1]:

$$H = -\sum_{<ij>} J_{\perp}(S^x_i S^x_j + S^y_i S^y_j) - \sum_{<ij>} J_z S^z_i S^z_j \tag{S2}$$

here $J_{\perp}$ is positive, J_z is negative, i.e. the system has a ferromagnetic inplane spin interaction, and an antiferromagnetic interaction between S^z. The pseudo-spin operators correspond to the boson operators in the following way:

$$S^+_i \sim b^{\dagger}_{i,2} b_{i,1}, \quad S^-_i \sim b^{\dagger}_{i,1} b_{i,2}, \quad S^z_i \sim \hat{n}_{i,2} - \hat{n}_{i,1}. \tag{S3}$$

The flux in the pseudo-spin Hamiltonian will turn on certain amount of frustration for the inplane components of the pseudo-spin, which is analogous to the fluxes seen by the orbital degrees of freedom derived for some of the graphene-based moiré systems [17, 32].

II. A PHENOMENOLOGICAL MODEL

The exact phase diagram of the Bose-Hubbard model (with fluxes), especially its fate at different fillings, deserve serious numerical studies. In this section we will discuss the physics of the system under doping away from $\nu_{ex} = 1$ with a simple phenomenological pseudo-spin model. Our phenomenological model Hamiltonian is motivated by the following observed phenomena of the exciton physics in TMD moiré heterostructure:

(1) Near exciton filling $\nu_{ex} = 1$, the pseudo-spin-1/2 degree of freedom of the exciton is in an inplane ferromagnet phase (labelled as the FM-xy phase); when doped with extra excitons, the pesudo-spin of the excitons is in the out-of-plane ferromagnet phase (labelled as the FM-z phase).

(2) Near filling $\nu_{ex} \sim 1.1$, the pseudo-spin is sensitive to a weak external Zeeman field: the pseudo-spin polarization $\langle S^z \rangle$ rapidly saturates under the external Zeeman field.

These phenomena can be qualitatively understood as a competition between the superexchange effect, and doping with extra excitons. First of all, as we have discussed, for a Mott insulator of bosons with pseudo-spin-1/2 internal degree of freedom, the superexchange which arises from virtual hopping of bosons will yield a *ferromagnetic* interaction between inplane components of the pseudo-spins (here we take $\phi_{ij} = 0$), and an *antiferromagnetic* interaction between S^z. It was also shown numerically that for a broad range of parameters for the XXZ spin-1/2 model on a triangular lattice, the system would have a FM-xy order [24] when the inplane superexchange is ferromagnetic. This is consistent with the observed inplane FM-xy phase at $\nu_{ex} = 1$.

In the following we argue that, under doping of extra bosons, the kinetic energy of the extra boson density would favor a FM-z order. This effect is based on the natural assumption that the intra-species onsite repulsion between the bosons is far stronger than the inter-species repulsion, and we also assume that the on-site repulsion interactions are far greater than the hopping amplitude of the excitons. The system would form a strong Mott insulator at filling

$\nu_{ex} = 1$, and in the strong repulsion limit all the pseudospin configurations are degenerate. But this degeneracy is lifted under doping with one extra boson, as for a doped boson to hop freely, it is favorable for all the “background" bosons at filling $\nu_{ex} = 1$ to have the same polarization of pseudo-spin S^z, say $S^z = +1$, while the extra boson has the opposite pseudo-spin $S^z = -1$, since as we assumed, the intra-species repulsion is far stronger than the inter-species repulsion. Hence the kinetic energy of the doped bosons would favor the system to have a net ferromagnetic polarization along the z direction. The physics here is to some extent analogous to the well-known doping-induced ferromagnet, i.e. the so-called Nagaoka ferromagnetism. We note here that the original Nagaoka's ferromagnet for the fermionic Hubbard model is isotropic in the spin space, but there is no SU(2) spin rotation symmetry for the pseudo-spin degree of freedom of the excitons. The SU(2) symmetry is broken explicitly by the difference between the intra-species and the inter-species repulsion, and the ferromagnetism for bosons under doping would favor a FM-z order.

In order to qualitatively capture the interpolation between the FM-xy and FM-z phases, we design the following phenomenological spin-1/2 Hamiltonian on the triangular moiré superlattice:

$$H = -\sum_{<ij>} J_\perp (S_i^x S_j^x + S_i^y S_j^y) - \sum_{<ij>,\ll ij \gg} J_z S_i^z S_j^z - \sum_i h S_i^z. \tag{S4}$$

The nearest-neighbor inplane interaction $J_\perp > 0$ (ferromagnetic) captures the effect of the superexchange at $\nu_{ex} = 1$. Since our experiments mostly probes the pseudo-spin physics of the exciton, we encapsulates the effects of the doped excitons into the renormalization of J_z. J_z would be antiferromagnetic ($J_z < 0$) at filling $\nu_{ex} = 1$, but it will gradually evolve into ferromagnetic interaction ($J_z > 0$) under doping. In our model Hamiltonian we include both first and second neighbor interactions for J_z, this is because if there were only nearest neighbor interaction between S^z, there would be a SU(2) symmetry at $J_\perp = J_z$, which as we commented before is unphysical. The mean field analysis to be presented here should be qualitatively independent of the microscopic details of the model.

We follow the standard procedure of mean field theory, and decompose the Hamiltonian as follows:

$$\begin{aligned} H_{MF} = & -\sum_{<ij>} J_\perp (\phi_x S_j^x + S_i^x \phi_x - \phi_x^2 + \phi_y S_j^y + S_i^y \phi_y - \phi_y^2) \\ & -\sum_{<ij>,\ll ij \gg} J_z (\phi_z S_j^z + S_i^z \phi_z - \phi_z^2) - \sum_i h S_i^z \end{aligned} \tag{S5}$$

ϕ_a can be physically viewed as the expectation value of S^a. The partition function of the mean field theory at finite temperature is given by

$$\begin{aligned} Z &= \mathrm{Tr}\{\exp(-\beta H_{MF})\} \\ &= \prod_i \mathrm{Tr}\{\exp\{\beta(6J_\perp\phi_x S^x + 6J_\perp\phi_y S^y + 12J_z\phi_z S^z + hS^z\}\} \\ &\times \exp\beta(-\sum_{<ij>} J_\perp\phi_x^2 + J_\perp\phi_y^2 - \sum_{<ij>,\ll ij\gg} J_z\phi_z^2) \\ &= \prod_i \cosh\beta\sqrt{(6J_\perp\phi_x)^2 + (6J_\perp\phi_y)^2 + (12J_z\phi_z + h)^2} \times \exp\big(-\beta(3NJ_\perp(\phi_x)^2 + 3NJ_\perp(\phi_y)^2 + 6NJ_z(\phi_z)^2)\big) \\ &= \Big(\cosh\beta\sqrt{(6J_\perp\phi_x)^2 + (6J_\perp\phi_y)y^2 + (12J_z\phi_z + h)^2}\exp\big(-\beta(3J_\perp\phi_x^2 + 3J_\perp\phi_y^2 + 6J_z\phi_z^2)\big)\Big)^N \end{aligned} \tag{S6}$$

The O(2) symmetry in the XY plane allows us to just consider ϕ_x and set $\phi_y = 0$ without loss of generality. The expression of the partition function then leads to the following free energy:

$$\begin{aligned} \frac{F}{N} &= -\frac{1}{\beta N}\ln Z \\ &= -\frac{1}{\beta}\ln\Big(\cosh\beta\sqrt{(6J_\perp\phi_x)^2 + (12J_z\phi_z + h)^2}\Big) + 3J_\perp\phi_x^2 + 6J_z\phi_z^2 \end{aligned} \tag{S7}$$

The variational condition would give us the following consistency equations to determine ϕ_a:

$$\frac{\partial F}{\partial \phi_x} = 0 \quad \rightarrow \quad \frac{6J_\perp\phi_x\tanh\Big(\beta\sqrt{(6J_\perp\phi_x)^2 + (12J_z\phi_z + h)^2}\Big)}{\sqrt{(6J_\perp\phi_x)^2 + (12J_z\phi_z + h)^2}} = \phi_x \tag{S8}$$

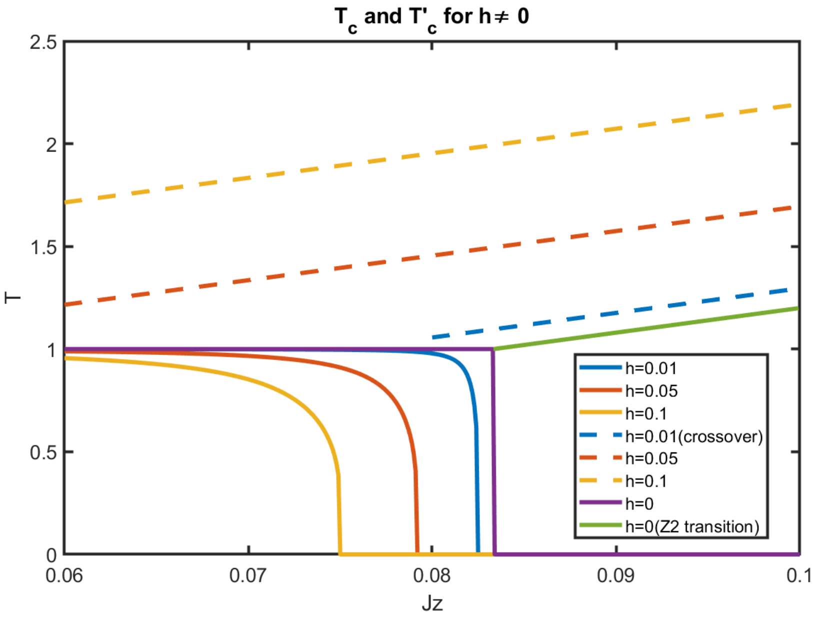

Supplementary Fig. 11: A plot of T_c and T'_c for different h.

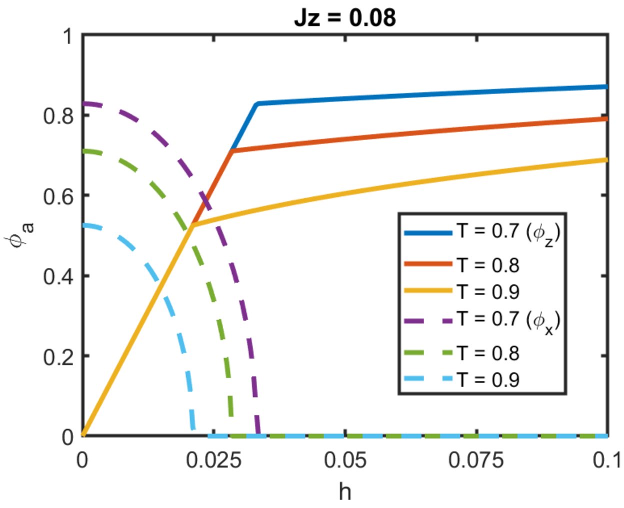

Supplementary Fig. 12: A plot of ϕ_z and ϕ_x vs h

$$\frac{\partial F}{\partial \phi_z} = 0 \quad \rightarrow \quad \frac{(12J_z\phi_z + h)\tanh\left(\beta\sqrt{(6J_\perp\phi_x)^2 + (12J_z\phi_z + h)^2}\right)}{\sqrt{(6J_\perp\phi_x)^2 + (12J_z\phi_z + h)^2}} = \phi_z \tag{S9}$$

The phase diagram of the mean field theory is plotted in Fig. 11. For small J_z, the system at zero temperature has a long range FM-xy order; at finite temperature the FM-xy order will become a quasi-long range order with power-law correlation. Within the mean field theory, the critical temperature of the FM-xy order is given by $T_c = 6J_\perp$, hence we choose $J_\perp = 1/6$ in the phase diagram to fix the energy scale of the superexchange interaction. While increasing J_z, there is a first order transition between the FM-xy and the FM-z order at low temperature.

A weak but finite Zeeman field h would favor the FM-z over FM-xy order. In Fig. 11 we can see that the FM-xy phase shrinks in the phase diagram under nonzero h. With a nonzero h, there is no longer a sharp transition between a FM-z order at low temperature, and a disordered phase at high temperature, but one can still define a crossover temperature T'_c which corresponds to $\langle S^z \rangle$ drops to $1/10$. A plot of T_c and T'_c on the $T - J_z$ plane for different values of h is shown in fig.11.

We also show how ϕ_x and ϕ_z evolve under increasing h (Fig. 12). J_z is chosen to be close to the phase boundary

between the FM-xy order and the FM-z. One can see that a relatively weak h can completely suppress the FM-xy order, and turn the system into the FM-z order, and the expectation value of S^z rapidly saturates. All these are consistent with what was observed experimentally.

The global phase diagram Eq. S1 may include various much more exotic phases, beyond the semiclassical ferromagnetic phases being discussed here. For example, if the system is not fixed at total filling $\nu_{ex} = 1$, each flavor of the exciton can be viewed as a hard-core boson with large U in Eq. S1, and the system may be viewed as an effective two-orbital spin-1/2 models as $\hat{n}_{i,\alpha} = 1, 0$ can be mapped to spin-up or down of a spin-1/2 degree of freedom. The entire system may also be viewed as a SU(4) spin model with certain anisotropy. SU(N) quantum magnets have attracted enormous interests since the early days of spin liquids [14, 19, 23, 18, 20, 21, 22, 8, 27], and the SU(4) quantum magnets are of particular interests as they can be engineered in transition metal oxides with spin and orbital degrees of freedom [15, 10], cold atom systems [26, 25, 5], and graphene based moiré systems [28, 31, 17, 30]. It was shown numerically that the system may support many possible exotic phases [11, 29, 4, 16, 7, 9, 33]. Also, the fluxes seen by the boson hoppingsin Eq. S1 will lead to spin-chirality terms in the effective Hamiltonian of the two-orbital spin-1/2 models, which may lead to topological orders according to various recent numerical works [6, 2, 3, 12, 13].

III. CLASSICAL $O(3)$ MODEL

Since the experiment is performed at finite temperature, we can also treat the spin vector S^a as a classical $O(3)$ vector with unit length. The Hamiltonian is still given by Eq. S4, but now S^a_i are components of a unit $O(3)$ classical vector.

The mean field decomposition proceeds as before and the mean field free energy is given as:

$$\begin{aligned}
Z &= \mathrm{Tr}\{\exp(-\beta H_{MF})\} \\
&= \prod_i \int D\vec{S} \exp\{\beta(6J_\perp \phi_x S^x + 6J_\perp \phi_y S^y + 12 J_z \phi_z S^z + h S^z\} \\
&\times \exp\beta(-\sum_{\langle ij\rangle} J_\perp \phi_x^2 + J_\perp \phi_y^2 - \sum_{\langle ij\rangle,\ll ij \gg} J_z \phi_z^2) \\
&= \left(\frac{4\pi}{\beta\sqrt{(6J_\perp\phi_x)^2 + (12J_z\phi_z + h)^2}} \sinh\beta\sqrt{(6J_\perp\phi_x)^2 + (12J_z\phi_z + h)^2} \exp\left(-\beta(3J_\perp\phi_x^2 + 3J_\perp\phi_y^2 + 6J_z\phi_z^2)\right)\right)^N
\end{aligned} \tag{S10}$$

We have again used the $O(2)$ symmetry to set $\phi_y = 0$. The free energy density is

$$f = -\frac{1}{\beta}\ln\left(\frac{4\pi}{\beta\sqrt{(6J_\perp\phi_x)^2 + (12J_z\phi_z + h)^2}} \sinh\beta\sqrt{(6J_\perp\phi_x)^2 + (12J_z\phi_z + h)^2}\right) + 3J_\perp\phi_x^2 + 6J_z\phi_z^2 \tag{S11}$$

The consistency equations for ϕ_x and ϕ_z then follow

$$\begin{aligned}
&6J_\perp\phi_x \frac{(6J_\perp\phi_x)^2 + (12J_z\phi_z + h)^2}{\sinh\beta\sqrt{(6J_\perp\phi_x)^2 + (12J_z\phi_z + h)^2}} \\
&\left(\frac{\cosh\beta\sqrt{(6J_\perp\phi_x)^2 + (12J_z\phi_z + h)^2}}{(6J_\perp\phi_x)^2 + (12J_z\phi_z + h)^2} - \frac{\sinh\beta\sqrt{(6J_\perp\phi_x)^2 + (12J_z\phi_z + h)^2}}{\beta((6J_\perp\phi_x)^2 + (12J_z\phi_z + h)^2)^{\frac{3}{2}}}\right) = \phi_x \qquad \text{(S12)} \\
&(12J_z\phi_z + h) \frac{(6J_\perp\phi_x)^2 + (12J_z\phi_z + h)^2}{\sinh\beta\sqrt{(6J_\perp\phi_x)^2 + (12J_z\phi_z + h)^2}} \\
&\left(\frac{\cosh\beta\sqrt{(6J_\perp\phi_x)^2 + (12J_z\phi_z + h)^2}}{(6J_\perp\phi_x)^2 + (12J_z\phi_z + h)^2} - \frac{\sinh\beta\sqrt{(6J_\perp\phi_x)^2 + (12J_z\phi_z + h)^2}}{\beta((6J_\perp\phi_x)^2 + (12J_z\phi_z + h)^2)^{\frac{3}{2}}}\right) = \phi_z. \qquad \text{(S13)}
\end{aligned}$$

The consistency equation can be solved numerically, and the physics is qualitatively the same as the quantum mean field theory presented in the previous section.

SUPPLEMENTARY REFERENCES

[1] Ehud Altman et al. "Phase diagram of two-component bosons on an optical lattice". In: *New Journal of Physics* 5.1 (Sept. 2003), p. 113. DOI: 10.1088/1367-2630/5/1/113. URL: https://dx.doi.org/10.1088/1367-2630/5/1/113.

[2] B. Bauer et al. “Chiral spin liquid and emergent anyons in a Kagome lattice Mott insulator”. In: *Nature Communications* 5.1 (Oct. 2014). DOI: 10.1038/ncomms6137. URL: https://doi.org/10.1038%2Fncomms6137.

[3] Ji-Yao Chen et al. “Non-Abelian chiral spin liquid in a quantum antiferromagnet revealed by an iPEPS study”. In: *Phys. Rev. B* 98 (18 Nov. 2018), p. 184409. DOI: 10.1103/PhysRevB.98.184409. URL: https://link.aps.org/doi/10.1103/PhysRevB.98.184409.

[4] Philippe Corboz et al. “Spin-Orbital Quantum Liquid on the Honeycomb Lattice”. In: *Phys. Rev. X* 2 (4 Nov. 2012), p. 041013. DOI: 10.1103/PhysRevX.2.041013. URL: https://link.aps.org/doi/10.1103/PhysRevX.2.041013.

[5] A. V. Gorshkov et al. “Two-orbital S U(N) magnetism with ultracold alkaline-earth atoms”. In: *Nature Physics* 6.4 (Feb. 2010), pp. 289–295. DOI: 10.1038/nphys1535. URL: https://doi.org/10.1038%2Fnphys1535.

[6] Martin Greiter and Ronny Thomale. “Non-Abelian Statistics in a Quantum Antiferromagnet”. In: *Phys. Rev. Lett.* 102 (20 May 2009), p. 207203. DOI: 10.1103/PhysRevLett.102.207203. URL: https://link.aps.org/doi/10.1103/PhysRevLett.102.207203.

[7] Michael Hermele, Victor Gurarie, and Ana Maria Rey. “Mott Insulators of Ultracold Fermionic Alkaline Earth Atoms: Underconstrained Magnetism and Chiral Spin Liquid”. In: *Phys. Rev. Lett.* 103 (13 Sept. 2009), p. 135301. DOI: 10.1103/PhysRevLett.103.135301. URL: https://link.aps.org/doi/10.1103/PhysRevLett.103.135301.

[8] Michael Hermele, T. Senthil, and Matthew P. A. Fisher. “Algebraic spin liquid as the mother of many competing orders”. In: *Phys. Rev. B* 72 (10 Sept. 2005), p. 104404. DOI: 10.1103/PhysRevB.72.104404. URL: https://link.aps.org/doi/10.1103/PhysRevB.72.104404.

[9] Anna Keselman et al. “Emergent Fermi Surface in a Triangular-Lattice SU(4) Quantum Antiferromagnet”. In: *Phys. Rev. Lett.* 125 (11 Sept. 2020), p. 117202. DOI: 10.1103/PhysRevLett.125.117202. URL: https://link.aps.org/doi/10.1103/PhysRevLett.125.117202.

[10] Kliment I Kugel’ and D I KhomskiÄ. “The Jahn-Teller effect and magnetism: transition metal compounds”. In: *Soviet Physics Uspekhi* 25.4 (Apr. 1982), p. 231. DOI: 10.1070/PU1982v025n04ABEH004537. URL: https://dx.doi.org/10.1070/PU1982v025n04ABEH004537.

[11] Y. Q. Li et al. “SU(4) Theory for Spin Systems with Orbital Degeneracy”. In: *Phys. Rev. Lett.* 81 (16 Oct. 1998), pp. 3527–3530. DOI: 10.1103/PhysRevLett.81.3527. URL: https://link.aps.org/doi/10.1103/PhysRevLett.81.3527.

[12] Zheng-Xin Liu et al. “Non-Abelian $S = 1$ chiral spin liquid on the kagome lattice”. In: *Phys. Rev. B* 97 (19 May 2018), p. 195158. DOI: 10.1103/PhysRevB.97.195158. URL: https://link.aps.org/doi/10.1103/PhysRevB.97.195158.

[13] Wei-Wei Luo et al. *Global quantum phase diagram and non-Abelian chiral spin liquid in a spin-3/2 square lattice antiferromagnet.* 2022. DOI: 10.48550/ARXIV.2212.14223. URL: https://arxiv.org/abs/2212.14223.

[14] J. Brad Marston and Ian Affleck. “Large-n limit of the Hubbard-Heisenberg model”. In: *Phys. Rev. B* 39 (16 June 1989), pp. 11538–11558. DOI: 10.1103/PhysRevB.39.11538. URL: https://link.aps.org/doi/10.1103/PhysRevB.39.11538.

[15] Swapan K. Pati, Rajiv R. P. Singh, and Daniel I. Khomskii. “Alternating Spin and Orbital Dimerization and Spin-Gap Formation in Coupled Spin-Orbital Systems”. In: *Phys. Rev. Lett.* 81 (24 Dec. 1998), pp. 5406–5409. DOI: 10.1103/PhysRevLett.81.5406. URL: https://link.aps.org/doi/10.1103/PhysRevLett.81.5406.

[16] Karlo Penc et al. “Quantum phase transition in the SU(4) spin-orbital model on the triangular lattice”. In: *Phys. Rev. B* 68 (1 July 2003), p. 012408. DOI: 10.1103/PhysRevB.68.012408. URL: https://link.aps.org/doi/10.1103/PhysRevB.68.012408.

[17] Hoi Chun Po et al. “Origin of Mott Insulating Behavior and Superconductivity in Twisted Bilayer Graphene”. In: *Phys. Rev. X* 8 (3 Sept. 2018), p. 031089. DOI: 10.1103/PhysRevX.8.031089. URL: https://link.aps.org/doi/10.1103/PhysRevX.8.031089.

[18] N. Read and Subir Sachdev. “Large-N expansion for frustrated quantum antiferromagnets”. In: *Phys. Rev. Lett.* 66 (13 Apr. 1991), pp. 1773–1776. DOI: 10.1103/PhysRevLett.66.1773. URL: https://link.aps.org/doi/10.1103/PhysRevLett.66.1773.

[19] N. Read and Subir Sachdev. “Some features of the phase diagram of the square lattice SU(N) antiferromagnet”. In: *Nuclear Physics B* 316.3 (1989), pp. 609–640. ISSN: 0550-3213. DOI: https://doi.org/10.1016/0550-3213(89)90061-8. URL: https://www.sciencedirect.com/science/article/pii/0550321389900618.

[20] N. Read and Subir Sachdev. “Spin-Peierls, valence-bond solid, and Néel ground states of low-dimensional quantum antiferromagnets”. In: *Phys. Rev. B* 42 (7 Sept. 1990), pp. 4568–4589. DOI: 10.1103/PhysRevB.42.4568. URL: https://link.aps.org/doi/10.1103/PhysRevB.42.4568.

[21] N. Read and Subir Sachdev. “Valence-bond and spin-Peierls ground states of low-dimensional quantum antiferromagnets”. In: *Phys. Rev. Lett.* 62 (14 Apr. 1989), pp. 1694–1697. DOI: 10.1103/PhysRevLett.62.1694. URL: https://link.aps.org/doi/10.1103/PhysRevLett.62.1694.

[22] Daniel S. Rokhsar. “Quadratic quantum antiferromagnets in the fermionic large-N limit”. In: *Phys. Rev. B* 42 (4 Aug. 1990), pp. 2526–2531. DOI: 10.1103/PhysRevB.42.2526. URL: https://link.aps.org/doi/10.1103/PhysRevB.42.2526.

[23] Subir Sachdev. "Kagomé- and triangular-lattice Heisenberg antiferromagnets: Ordering from quantum fluctuations and quantum-disordered ground states with unconfined bosonic spinons". In: *Phys. Rev. B* 45 (21 June 1992), pp. 12377–12396. DOI: 10.1103/PhysRevB.45.12377. URL: https://link.aps.org/doi/10.1103/PhysRevB.45.12377.

[24] Fa Wang, Frank Pollmann, and Ashvin Vishwanath. "Extended Supersolid Phase of Frustrated Hard-Core Bosons on a Triangular Lattice". In: *Phys. Rev. Lett.* 102 (1 Jan. 2009), p. 017203. DOI: 10.1103/PhysRevLett.102.017203. URL: https://link.aps.org/doi/10.1103/PhysRevLett.102.017203.

[25] Congjun Wu. "Competing Orders in One-Dimensional Spin-3/2 Fermionic Systems". In: *Phys. Rev. Lett.* 95 (26 Dec. 2005), p. 266404. DOI: 10.1103/PhysRevLett.95.266404. URL: https://link.aps.org/doi/10.1103/PhysRevLett.95.266404.

[26] Congjun Wu, Jiang-ping Hu, and Shou-cheng Zhang. "Exact SO(5) Symmetry in the Spin-3/2 Fermionic System". In: *Phys. Rev. Lett.* 91 (18 Oct. 2003), p. 186402. DOI: 10.1103/PhysRevLett.91.186402. URL: https://link.aps.org/doi/10.1103/PhysRevLett.91.186402.

[27] Cenke Xu. "Liquids in multiorbital SU(N) magnets made up of ultracold alkaline-earth atoms". In: *Phys. Rev. B* 81 (14 Apr. 2010), p. 144431. DOI: 10.1103/PhysRevB.81.144431. URL: https://link.aps.org/doi/10.1103/PhysRevB.81.144431.

[28] Cenke Xu and Leon Balents. "Topological Superconductivity in Twisted Multilayer Graphene". In: *Phys. Rev. Lett.* 121 (8 Aug. 2018), p. 087001. DOI: 10.1103/PhysRevLett.121.087001. URL: https://link.aps.org/doi/10.1103/PhysRevLett.121.087001.

[29] Cenke Xu and Congjun Wu. "Resonating plaquette phases in SU(4) Heisenberg antiferromagnet". In: *Phys. Rev. B* 77 (13 Apr. 2008), p. 134449. DOI: 10.1103/PhysRevB.77.134449. URL: https://link.aps.org/doi/10.1103/PhysRevB.77.134449.

[30] Yi-Zhuang You and Ashvin Vishwanath. "Superconductivity from valley fluctuations and approximate SO(4) symmetry in a weak coupling theory of twisted bilayer graphene". In: *npj Quantum Materials* 4.1 (Apr. 2019). DOI: 10.1038/s41535-019-0153-4. URL: https://doi.org/10.1038%2Fs41535-019-0153-4.

[31] Noah F. Q. Yuan and Liang Fu. "Model for the metal-insulator transition in graphene superlattices and beyond". In: *Phys. Rev. B* 98 (4 July 2018), p. 045103. DOI: 10.1103/PhysRevB.98.045103. URL: https://link.aps.org/doi/10.1103/PhysRevB.98.045103.

[32] Ya-Hui Zhang and T. Senthil. "Bridging Hubbard model physics and quantum Hall physics in trilayer graphene/$h-$BN moiré superlattice". In: *Phys. Rev. B* 99 (20 May 2019), p. 205150. DOI: 10.1103/PhysRevB.99.205150. URL: https://link.aps.org/doi/10.1103/PhysRevB.99.205150.

[33] Ya-Hui Zhang, D. N. Sheng, and Ashvin Vishwanath. "SU(4) Chiral Spin Liquid, Exciton Supersolid, and Electric Detection in Moiré Bilayers". In: *Phys. Rev. Lett.* 127 (24 Dec. 2021), p. 247701. DOI: 10.1103/PhysRevLett.127.247701. URL: https://link.aps.org/doi/10.1103/PhysRevLett.127.247701.
